# Association Between the Planetary Health Diet Index and Chronic Kidney Disease Prevalence and Mortality: An Analysis of NHANES 2005–2018

**DOI:** 10.1002/fsn3.71402

**Published:** 2025-12-26

**Authors:** Yufei Ming, Zhonghua Sun, Minyi Tao, Jiahui Yang, Shuyan Li, Xinyu Tao, Haiyang Wang, Chen Qu, Zhengxia Liu

**Affiliations:** ^1^ Department of Geriatrics The Second Affiliated Hospital of Nanjing Medical University Nanjing China; ^2^ Medical School of Nanjing University Nanjing China

**Keywords:** albuminuria, chronic kidney disease, kidney function, mortality, NHANES, planetary health diet index

## Abstract

Diet is an important determinant of chronic kidney disease (CKD). The EAT–Lancet Commission proposed the Planetary Health Diet (PHD) in 2019, and the Planetary Health Diet Index (PHDI) was developed in 2021 to quantify adherence. The index includes 16 food‐group components (e.g., nuts, red meat). However, few studies have assessed associations between the PHDI and its subcomponents with CKD prevalence and CKD‐related mortality. We used survey‐weighted multivariable logistic regression to assess associations between the PHDI and CKD prevalence, kidney function (estimated glomerular filtration rate, eGFR), and albuminuria; Cox proportional hazards models were used to evaluate CKD‐related mortality. Potential nonlinear dose–response was evaluated using restricted cubic splines and segmented regression. Predictive discrimination was quantified using time‐dependent receiver operating characteristic (ROC) curves. We analyzed associations between PHDI subcomponents and CKD prevalence and CKD‐related mortality. Among 31,703 adults, a higher PHDI was associated with lower CKD prevalence and CKD‐related mortality. Associations were nonlinear with a threshold. The conventional ROC curve exhibited good discrimination for CKD prevalence, and the time‐dependent ROC curve showed robust prognostic discrimination for CKD‐related mortality. A higher PHDI was correlated with lower albuminuria levels and less impaired kidney function; nuts, vegetables, and legumes exhibited consistent protective effects. Greater PHDI adherence was linked to lower CKD prevalence, better kidney function, and reduced CKD‐related mortality. Restricted cubic spline analyses revealed nonlinear patterns, with prevalence inflection at ~64 and a mortality threshold of ~47. Clinically, these thresholds may guide dietary recommendations, emphasizing increased intake of nuts, vegetables, and legumes.

## Graphical abstract

Adherence to the Planetary Health Diet Index (PHDI) was associated with lower CKD prevalence, improved kidney function, and reduced CKD‐related mortality. Protective effects emerged only after exceeding specific PHDI thresholds. Nuts and seeds, non‐starchy vegetables, and legumes consistently reduced CKD prevalence, and nut intake additionally lowered CKD‐related mortality.

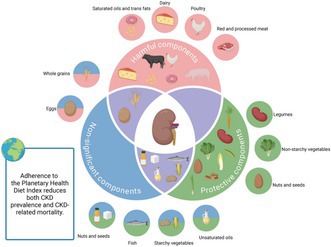

## 1 Introduction

Chronic kidney disease (CKD) affects approximately one in ten adults worldwide (GBD Chronic Kidney Disease Collaboration 2020; Hill et al. 2016; Stevens et al. 2024). Beyond increasing the risk of cardiovascular events and premature mortality, CKD contributes to fatigue, sleep disturbances, depression, and a diminished capacity for independent living or employment (GBD Chronic Kidney Disease Collaboration 2020; Stevens et al. 2024; Lyons 2024; Palmer et al. 2013; Fletcher et al. 2022). Treatment options available for CKD are often limited and financially burdensome, whereas lifestyle interventions, particularly dietary modifications, may offer more accessible and cost‐effective preventive strategies (Luyckx et al. 2018; Carrero et al. 2020; Kelly et al. 2017).

Diet is closely involved in CKD development and progression. Saturated fat and processed meat intake are related to faster renal decline, whereas consumption patterns rich in vegetables, fruits, whole grains, and unsaturated fats are associated with slower progression (Carrero et al. 2020; Kelly et al. 2021; Lew et al. 2017; Hu et al. 2019; Cai et al. 2021). Cohort studies have also associated plant‐based patterns with lower CKD incidence and less albuminuria (Cai et al. 2021; He et al. 2021; Narasaki et al. 2023).

Grounded in the EAT‐Lancet recommendations proposed in 2019, the Planetary Health Diet Index (PHDI) operationalizes a diet that is nutritionally adequate and environmentally sustainable (Willett et al. 2019; Cacau et al. 2021). On the basis of these recommendations, Cacau et al. (2021) refined the dietary targets into 16 scored components, yielding a total score ranging from 0 to 150 points (Cacau et al. 2021). To ensure compatibility with the National Health and Nutrition Examination Survey (NHANES) food classification system and the Food Patterns Equivalents Database (FPED), several NHANES‐based studies have adapted the PHDI by modifying its component structure, resulting in a total score of 0–140 points (Huang et al. 2024; Qi et al. 2025; Yang et al. 2025; Chen et al. 2025). In the present study, we adopted this adapted 0–140‐point PHDI scoring system to ensure full compatibility with NHANES dietary data and FPED classifications. The detailed scoring criteria are presented in Table S1. The PHDI subcomponents comprise adequacy components (whole grains, whole fruits, non‐starchy vegetables, nuts and seeds, non‐soy legumes, soy foods, and unsaturated oils) and moderation components (starchy vegetables, dairy, red and processed meats, poultry, eggs, fish, saturated + trans fats, and added sugar + fruit juice). Previous studies have shown that higher PHDI scores correspond to a lower likelihood of developing metabolic disorders including metabolic syndrome, type 2 diabetes, and cardiovascular disease (Qiu et al. 2025; Frank, Jaacks, Avery, et al. 2024; Tang, Zhang, et al. 2025; Nair et al. 2025). Among individuals with CKD, greater adherence to the PHDI is associated with reduced risks of sarcopenia (Chen et al. 2025; Mansouri et al. 2025).

While indices such as the Healthy Eating Index (HEI), Mediterranean Diet Score, and Alternate Healthy Eating Index (AHEI) primarily assess nutritional quality, the PHDI additionally incorporates environmental sustainability, thereby echoing contemporary global dietary agendas (Cacau et al. 2021; Conrad et al. 2025; Frank, Jaacks, Meyer, et al. 2024). Previous studies have associated increased adherence to the PHDI with a decelerated pace of cognitive decline and enhanced psychological well‐being (Tang, Yu, et al. 2025; Jiang et al. 2025), suggesting that the PHDI may serve as a more holistic tool for evaluating dietary quality in relation to both individual and planetary health.

We used the NHANES database to analyze US adults. Our objective was to determine how adherence to the PHDI is related to CKD prevalence and CKD‐related mortality. We also explored which PHDI components were most strongly associated with CKD risk and whether the PHDI could predict long‐term kidney‐related mortality.

## 2 Methods

### 2.1 Study Population

To ensure U.S. national representativeness, NHANES employs a probability‐based, multistage, stratified sampling design to recruit civilian, community‐dwelling adults from the 50 states and Washington, D.C., in biennial cycles under Centers for Disease Control and Prevention (CDC) oversight. Data collection includes structured household interviews, clinic‐style physical examinations, and laboratory measurements conducted in Mobile Examination Centers (MECs), all governed by standardized protocols (Centers for Disease Control and Prevention (CDC) 2017; Centers for Disease Control and Prevention (CDC) 2018).

The analytic cohort included community‐dwelling, nonpregnant adults (≥ 20 years) enrolled in seven NHANES cycles from 2005 through 2018 who completed two valid 24‐h dietary recalls and had complete data on kidney function and relevant covariates. Participants with implausible total energy intake (< 500 or > 8000 kcal/day) were excluded. In total, 31,703 individuals contributed to the CKD prevalence analysis, including 5769 with CKD. Among those with CKD, 5764 had complete mortality linkage and were included in the CKD‐related mortality analysis.

### 2.2 Dietary Data

Two nonconsecutive 24‐h dietary recalls formed the basis of dietary assessment. The first recall was conducted in person at MECs on the examination day, and the second recall was conducted by telephone 3–10 days later. Both recalls were administered by trained interviewers using the U.S. Department of Agriculture (USDA) Automated Multiple‐Pass Method (AMPM) (Centers for Disease Control and Prevention (CDC) 2017; Centers for Disease Control and Prevention (CDC) 2018; Ettienne‐Gittens et al. 2013). Computation of nutrient totals and food‐group equivalents relied on the Food and Nutrient Database for Dietary Studies (FNDDS).

Dietary items were harmonized into FPED food‐group equivalents using the 2017–2018 FPED (U.S. Department of Agriculture 2020), which classifies over 7000 foods into 37 components aligned with U.S. dietary guidelines. However, consistent with the PHDI framework, 35 components—excluding the two fatty‐acid ratio components—were used in this analysis. Each component is expressed in appropriate units: cup equivalents (e.g., fruits, vegetables, dairy), ounce equivalents (e.g., grains, protein foods), teaspoon equivalents (e.g., added sugars), or gram equivalents (e.g., solid fats, oils), and alcoholic drinks as number of drinks. Dairy intake was quantified as whole milk equivalents to align with the nutritional and environmental framework of the PHDI (Cacau et al. 2021; U.S. Department of Agriculture 2020).

When two recalls were present, intake was averaged; otherwise, the day 1 recall was used as the intake estimate (Centers for Disease Control and Prevention (CDC) 2017; Centers for Disease Control and Prevention (CDC) 2018).

### 2.3 Measurement of the PHDI

To ensure compatibility with NHANES and the FPED system (U.S. Department of Agriculture 2020), we modified the scoring structure of the original 16‐component PHDI (Cacau et al. 2021). Specifically, the legumes component was divided into non‐soy legumes and soy foods, each contributing 50% of the total legumes score; added sugar and fruit juice were combined into a single moderation component following FPED classification. These adaptations resulted in a 14‐component PHDI with a total score ranging from 0 to 140 points. The 14 components were further classified as adequacy or moderation components. Adequacy components were assigned higher scores with higher intakes, with the maximum score being assigned at the midpoint of the EAT–Lancet recommended intake range (the average of the lower and upper bounds). For moderation components, lower intakes received higher scores, whereas intakes at or above the EAT–Lancet upper limit were assigned zero points. Intakes between the two thresholds were assigned proportionally using linear interpolation (Willett et al. 2019; Cacau et al. 2021). To maintain compatibility with EAT–Lancet targets, intakes were primarily expressed in grams. For dairy, we translated grams into serving‐equivalents according to FPED (with 1 cup of whole milk defined as 245 g) (U.S. Department of Agriculture 2020).

Table S1 summarizes the scoring criteria for all 14 PHDI components, detailing the intake thresholds defining minimum (0 points) and maximum (10 points) scores for each adequacy and moderation component.

### 2.4 Outcome Definitions: Diagnosis of CKD and Mortality Classification

In alignment with prior analyses (Guo et al. 2022; Kang et al. 2021) and Kidney Disease: Improving Global Outcomes (KDIGO) guidance (Stevens et al. 2024), CKD was defined as estimated glomerular filtration rate (eGFR) < 60 mL/min/1.73 m^2^ or urinary albumin–creatinine ratio (ACR) ≥ 30 mg/g.

We used the race‐free CKD‐EPI creatinine equation (2021) with age and sex adjustments to estimate eGFR; no race coefficient was applied (Stevens et al. 2024; Inker et al. 2021). Serum creatinine and urinary albumin/creatinine levels were obtained from NHANES laboratory data, which were collected once during the MECs visit on the examination day.

Each death in the NHANES Linked Mortality Files was coded using UCOD_LEADING according to the International Classification of Diseases, Tenth Revision (ICD‐10), which classifies deaths into ten primary categories (codes “001”–“010”) in the 2019 public‐use dataset. In this study, deaths with UCOD_LEADING = “009” (nephritis, nephrotic syndrome, and nephrosis) were defined as CKD‐related mortality.

### 2.5 Covariates

Covariates included demographic, socioeconomic, lifestyle, and clinical variables known to be associated with CKD (Grobman et al. 2025; Vart et al. 2020; Xie et al. 2025). Demographic characteristics comprised participants' age, sex, and race/ethnicity, categorized as non‐Hispanic White, non‐Hispanic Black, Mexican American, other Hispanic, and all other racial groups. Socioeconomic indicators were determined through educational attainment (< 9th grade, 9–11th grade, high school graduate, some college or associate degree, and college graduate or above) as well as the poverty–income ratio (PIR). Lifestyle and clinical variables included body mass index (BMI), smoking behavior (never, former, or current), diabetes status (defined by self‐report, fasting plasma glucose ≥ 126 mg/dL, HbA1c ≥ 6.5%, a 2‐h Oral Glucose Tolerance Test (OGTT) result ≥ 200 mg/dL when available, or current use of glucose‐lowering medication), hypertension (identified by mean blood pressure ≥ 140/90 mmHg, self‐reported physician diagnosis, or the use of antihypertensive drugs), and hypercholesterolemia (total cholesterol ≥ 200 mg/dL, self‐report of diagnosis, or treatment with lipid‐lowering agents). All anthropometric and biochemical measurements were obtained at the MECs by trained staff following standardized protocols.

### 2.6 Statistical Analysis

All analyses were conducted with R software (version 4.4.2). To appropriately represent the probability‐based, multistage, stratified sampling design of NHANES, the official CDC guidelines were followed, incorporating sampling weights, strata, and primary sampling units into all estimations. Continuous variables were reported as weighted means with standard deviations (SD), while categorical variables were presented as weighted proportions. Differences between groups were examined using survey‐adjusted linear regression for continuous outcomes and survey‐adjusted chi‐square tests for categorical outcomes.

Associations between the PHDI and kidney outcomes were estimated with survey‐weighted multivariable logistic regression models. Outcomes of interest included: (1) prevalent CKD, (2) impaired kidney function (eGFR < 60 mL/min/1.73 m^2^), and (3) albuminuria (urinary ACR ≥ 30 mg/g). PHDI scores were analyzed both as a continuous variable and as a binary variable, dichotomized at the median. Four regression models were sequentially specified, with progressive adjustment for sociodemographic, lifestyle, and clinical covariates. To explore potential nonlinear dose–response relationships, restricted cubic spline (RCS) models with three knots were fitted. Segmented regression was further applied to detect breakpoints along the PHDI–CKD association curve. We also examined the associations of individual PHDI components with CKD prevalence using fully adjusted survey‐weighted logistic regression models (model 3: comprehensively adjusted for sociodemographic, lifestyle, and clinical characteristics) with the corresponding estimates summarized in Figure S1.

For CKD‐related mortality, Cox proportional hazards models were constructed using the same adjustment strategy. Kaplan–Meier survival curves and log‐rank tests were performed to compare survival probabilities across PHDI strata. Furthermore, time‐dependent Receiver operating characteristic (ROC) curves were generated to evaluate the discriminative ability of PHDI‐based models at 3‐year and 5‐year follow‐up. All statistical tests were two‐sided, and results with *p* < 0.05 were considered statistically significant.

## 3 Results

### 3.1 Study Population Characteristics

**FIGURE 1:**
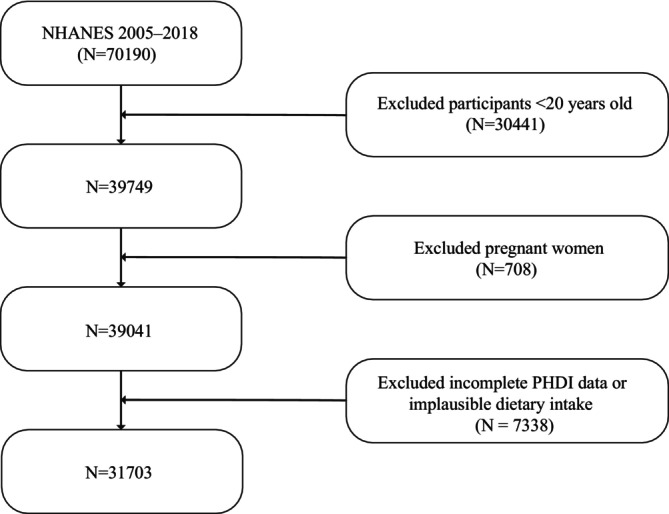
Flowchart of participant selection from NHANES 2005–2018. NHANES, National Health and Nutrition Examination Survey; PHDI, planetary health diet index.

In total, 31,703 U.S. adults aged 20 years and older were analyzed; among them, 5769 participants (18.2%) met the definition of CKD (Table 1). As shown in Figure 1, the participant selection process began with the NHANES 2005–2018 cohort, and the flowchart illustrates the detailed screening steps. Compared with those without CKD, participants with CKD tended to be older, had lower eGFR, and carried a higher burden of comorbidities, particularly diabetes, hypertension, and hypercholesterolemia. They also had lower education levels, higher obesity rates, and were more likely to be current smokers.

**TABLE 1:** Initial characteristics of the study participants.

Variables	Total	Non‐CKD	CKD	*p*
	(*N* = 31,703)	(*N* = 25,934)	(*N* = 5769)	
Age (year)	49.90 ± 17.64	46.91 ± 16.50	63.31 ± 16.28	**< 0.001**
Gender				
Female	16,111 (50.82%)	13,106 (50.54%)	3005 (52.09%)	**0.034**
Male	15,592 (49.18%)	12,828 (49.46%)	2764 (47.91%)	
Race				
Mexican American	5040 (15.90%)	4270 (16.46%)	770 (13.35%)	**< 0.001**
Other Hispanic	3053 (9.63%)	2596 (10.01%)	457 (7.92%)	
Non‐Hispanic White	13,772 (43.44%)	10,928 (42.14%)	2844 (49.30%)	
Non‐Hispanic Black	6514 (20.55%)	5267 (20.31%)	1247 (21.62%)	
Other race	3324 (10.48%)	2873 (11.08%)	451 (7.82%)	
PIR				
< 1.3	9823 (30.98%)	7884 (30.40%)	1939 (33.61%)	**< 0.001**
1.3–3.5	12,030 (37.95%)	9559 (36.86%)	2471 (42.83%)	
≥ 3.5	9850 (31.07%)	8491 (32.74%)	1359 (23.56%)	
Education level				
< 9th Grade	3308 (10.43%)	2443 (9.42%)	865 (14.99%)	**< 0.001**
9–11th Grade	4389 (13.84%)	3461 (13.35%)	928 (16.09%)	
High School Graduate	7262 (22.91%)	5852 (22.56%)	1410 (24.44%)	
Some College/AA Degree	9371 (29.56%)	7800 (30.08%)	1571 (27.23%)	
College Graduate or Above	7373 (23.26%)	6378 (24.59%)	995 (17.25%)	
BMI Category				
Normal weight	8983 (28.33%)	7611 (29.35%)	1372 (23.78%)	**< 0.001**
Overweight	10,558 (33.30%)	8725 (33.64%)	1833 (31.77%)	
Obese	12,162 (38.36%)	9598 (37.01%)	2564 (44.44%)	
Smoking Status				
Never smoker	17,505 (55.22%)	14,589 (56.25%)	2916 (50.55%)	**< 0.001**
Former smoker	6411 (20.22%)	5466 (21.08%)	945 (16.38%)	
Current smoker	7787 (24.56%)	5879 (22.67%)	1908 (33.07%)	
Hypertension				
No	20,228 (63.80%)	18,108 (69.82%)	2120 (36.75%)	**< 0.001**
Yes	11,475 (36.20%)	7826 (30.18%)	3649 (63.25%)	
Diabetes				
No	26,270 (82.86%)	22,682 (87.46%)	3588 (62.19%)	**< 0.001**
Yes	5433 (17.14%)	3252 (12.54%)	2181 (37.81%)	
Hyperlipidemia				
No	19,927 (62.86%)	17,095 (65.92%)	2832 (49.09%)	**< 0.001**
Yes	11,776 (37.14%)	8839 (34.08%)	2937 (50.91%)	
PHDI Score	64.37 ± 13.10	64.27 ± 13.31	64.81 ± 12.13	**0.003**
eGFR (mL/min/1.73 m^2^)	93.42 ± 23.79	98.42 ± 19.07	70.95 ± 29.28	**< 0.001**
ACR (mg/g)	46.32 ± 349.90	8.13 ± 5.62	217.97 ± 797.96	**< 0.001**
PHDI Quartiles				
Q1	21,939 (69.20%)	17,967 (69.28%)	3972 (68.85%)	**0.002**
Q2	9764 (30.80%)	7967 (30.72%)	1797 (31.15%)	

*Note:* Age, estimated glomerular filtration rate (eGFR), albumin–creatinine ratio (ACR), and PHDI score are continuous variables presented as mean ± standard deviation (SD). All other variables, including PHDI median‐based groups, are categorical and presented as frequencies (percentages). Bold values indicate *p* < 0.05.

Abbreviations: CKD, chronic kidney disease; PHDI, planetary health diet index; PIR, poverty income ratio.

Table 2 presents baseline characteristics of participants with CKD according to all‐cause mortality status. Relative to survivors, individuals who died were older (mean age 73.5 vs. 59.1 years, *p* < 0.001), had a higher proportion of men (55.8% vs. 44.7%, *p* < 0.001), and were more frequently non‐Hispanic White (65.0% vs. 42.9%, *p* < 0.001). Decedents also showed lower PHDI scores (64.1 vs. 65.1, *p* = 0.003), reduced eGFR (57.0 vs. 76.7 mL/min/1.73 m^2^, *p* < 0.001), and a higher prevalence of hypertension (72.9% vs. 59.3%, *p* < 0.001) and diabetes (43.0% vs. 35.7%, *p* < 0.001). Current smoking was likewise more common among those who died (42.5% vs. 29.2%; most comparisons *p* < 0.001; *p* for PHDI = 0.003).

**TABLE 2:** Baseline characteristics of patients with CKD.

Variables	Total	Non‐death	Death	*p*
	(*N* = 5764)	(*N* = 4088)	(*N* = 1676)	
Age (year)	63.31 ± 16.28	59.14 ± 16.55	73.48 ± 9.87	**< 0.001**
Gender				
Female	3002 (52.08%)	2261 (55.31%)	741 (44.21%)	**< 0.001**
Male	2762 (47.92%)	1827 (44.69%)	935 (55.79%)	
Race				
Mexican American	770 (13.36%)	638 (15.61%)	132 (7.88%)	**< 0.001**
Other Hispanic	456 (7.91%)	384 (9.39%)	72 (4.30%)	
Non‐Hispanic White	2844 (49.34%)	1755 (42.93%)	1089 (64.98%)	
Non‐Hispanic Black	1246 (21.62%)	929 (22.73%)	317 (18.91%)	
Other race	448 (7.77%)	382 (9.34%)	66 (3.94%)	
PIR				
< 1.3	1936 (33.59%)	1348 (32.97%)	588 (35.08%)	**< 0.001**
1.3–3.5	2471 (42.87%)	1690 (41.34%)	781 (46.60%)	
≥ 3.5	1357 (23.54%)	1050 (25.68%)	307 (18.32%)	
Education level				
< 9th Grade	863 (14.97%)	563 (13.77%)	300 (17.90%)	**< 0.001**
9–11th Grade	927 (16.08%)	599 (14.65%)	328 (19.57%)	
High School Graduate	1410 (24.46%)	979 (23.95%)	431 (25.72%)	
Some College/AA Degree	1570 (27.24%)	1171 (28.64%)	399 (23.81%)	
College Graduate or Above	994 (17.25%)	776 (18.98%)	218 (13.01%)	
BMI Category				
Normal weight	1372 (23.80%)	877 (21.45%)	495 (29.53%)	**< 0.001**
Overweight	1829 (31.73%)	1286 (31.46%)	543 (32.40%)	
Obese	2563 (44.47%)	1925 (47.09%)	638 (38.07%)	
Smoking Status				
Never smoker	2912 (50.52%)	2213 (54.13%)	699 (41.71%)	**< 0.001**
Former smoker	945 (16.39%)	680 (16.63%)	265 (15.81%)	
Current smoker	1907 (33.08%)	1195 (29.23%)	712 (42.48%)	
Hypertension				
No	2120 (36.78%)	1665 (40.73%)	455 (27.15%)	**< 0.001**
Yes	3644 (63.22%)	2423 (59.27%)	1221 (72.85%)	
Diabetes				
No	3586 (62.21%)	2630 (64.33%)	956 (57.04%)	**< 0.001**
Yes	2178 (37.79%)	1458 (35.67%)	720 (42.96%)	
Hyperlipidemia				
No	2829 (49.08%)	2080 (50.88%)	749 (44.69%)	**< 0.001**
Yes	2935 (50.92%)	2008 (49.12%)	927 (55.31%)	
PHDI Score	64.80 ± 12.13	65.09 ± 12.50	64.09 ± 11.15	**0.003**
PHDI Quartiles				
Q1	2885 (50.05%)	2010 (49.2%)	875 (52.2%)	**0.002**
Q2	2879 (49.95%)	2078 (50.8%)	801 (47.8%)	

*Note:* Age and PHDI score are continuous variables presented as mean ± standard deviation (SD). All other variables, including PHDI median‐based groups, are categorical and presented as frequencies (percentages). Bold values indicate *p* < 0.05.

Abbreviations: CKD, chronic kidney disease; PHDI, planetary health diet index; PIR, poverty income ratio.

### 3.2 Association Between PHDI and CKD Prevalence

#### 3.2.1 Logistic Regression Results

We applied survey‐weighted multivariable logistic regression to examine the association between PHDI and prevalent CKD (Table 3). In Model 0 without covariate adjustment, each one‐point increase in PHDI was linked to higher odds of CKD (OR = 1.003, 95% CI 1.001–1.005; *p* < 0.001), which was likely attributable to confounding by age and sex. After adding age and sex in Model 1, the association reversed direction and became inverse (OR = 0.990, 95% CI 0.987–0.992; *p* < 0.001). The negative association persisted with further adjustments, remaining evident in Model 2 after inclusion of sociodemographic factors (OR = 0.993, 95% CI 0.990–0.995; *p* < 0.001) and in the fully adjusted Model 3 that additionally accounted for clinical and lifestyle variables (OR = 0.992, 95% CI 0.990–0.995; *p* < 0.001). Overall, once major confounders were controlled for, higher PHDI scores consistently corresponded to lower CKD prevalence.

**TABLE 3:** Weighted logistic regression analysis on the associations between the Planetary Health Diet Index (PHDI) and CKD, albuminuria, and low eGFR.

	CKD		Albuminuria		Low eGFR	
	OR (95% CI)	*p*	OR (95% CI)	*p*	OR (95% CI)	*p*
Model 1						
PHDI Score	0.990 (0.987, 0.992)	**< 0.001**	0.992 (0.989, 0.994)	**< 0.001**	0.986 (0.983, 0.990)	**< 0.001**
Q1	ref		ref		ref	
Q2	0.821 (0.771, 0.874)	**< 0.001**	0.831 (0.775, 0.890)	**< 0.001**	0.760 (0.695, 0.831)	**< 0.001**
*p* for trend		**< 0.001**		**< 0.001**		**< 0.001**
Model 2						
PHDI Score	0.993 (0.990, 0.995)	**< 0.001**	0.994 (0.991, 0.997)	**< 0.001**	0.986 (0.982, 0.989)	**< 0.001**
Q1	ref		ref		ref	
Q2	0.865 (0.812, 0.922)	**< 0.001**	0.876 (0.817, 0.940)	**< 0.001**	0.758 (0.693, 0.829)	**< 0.001**
*p* for trend		**< 0.001**		**< 0.001**		**< 0.001**
Model 3						
PHDI Score	0.992 (0.990, 0.995)	**< 0.001**	0.994 (0.991, 0.997)	**< 0.001**	0.986 (0.983, 0.990)	**< 0.001**
Q1	ref		ref		ref	
Q2	0.844 (0.791, 0.900)	**< 0.001**	0.860 (0.800, 0.924)	**< 0.001**	0.761 (0.695, 0.834)	**< 0.001**
*p* for trend		**< 0.001**		**< 0.001**		**< 0.001**

*Note:* Model 1: Adjusted for age and sex. Model 2: Additionally adjusted for race/ethnicity, education level, income level, BMI category, and smoking status. Model 3: Additionally adjusted for hypertension, diabetes, and hypercholesterolemia. PHDI was modeled both continuously (per 1‐point increase) and as a binary variable (Q2 vs. Q1). Results are presented as odds ratios (ORs) with 95% confidence intervals (CIs). Bold values indicate statistically significant associations (*p* < 0.05).

Abbreviations: CI, confidence interval; CKD, chronic kidney disease; OR, odds ratio; PHDI, planetary health diet index.

#### 3.2.2 Nonlinear Association Between PHDI and CKD Prevalence

**FIGURE 2:**
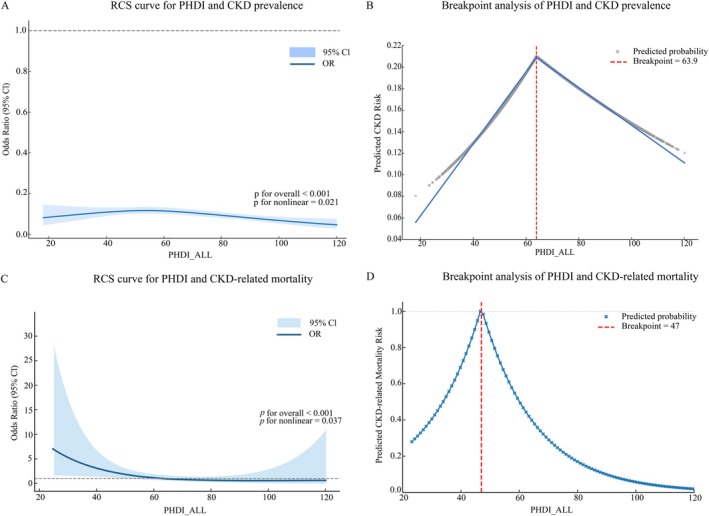
Nonlinear associations of the Planetary Health Diet Index (PHDI) with CKD outcomes. (A) RCS analysis of CKD prevalence (*p* for overall < 0.001; *p* for non‐linearity = 0.021). (B) Segmented regression for CKD prevalence with breakpoint at PHDI = 63.9. (C) RCS analysis of CKD‐related mortality (*p* for overall < 0.001; *p* for non‐linearity = 0.037). (D) Segmented regression for CKD‐related mortality with breakpoint at PHDI = 47. RCS, restricted cubic spline; CKD, chronic kidney disease; PHDI, Planetary Health Diet Index.

Analyses based on generalized additive models (GAMs) and RCS suggested a nonlinear association between PHDI and CKD prevalence. Figure 2A displays the RCS results for the relationship between PHDI and CKD prevalence. The risk of CKD initially increased with PHDI up to approximately PHDI = 60 (*χ* ^2^ = 10.31, *p* = 0.006). Beyond this threshold, higher PHDI scores were associated with a protective effect, with *p* for nonlinear = 0.021, indicating a statistically significant nonlinear relationship. Additionally, *p* for overall < 0.001 suggests that the overall model demonstrates a highly significant association.

Segmented regression analysis further characterized the nonlinear pattern identified in the spline model and is presented in Figure 2B. The model detected a clear breakpoint at a PHDI score of approximately 64 (63.9). Below this point, no significant trend was observed (OR = 1.000, *p* = 0.948), but beyond this threshold, each one‐point increase in PHDI was associated with an approximately 1.3% reduction in CKD odds (OR = 0.987, *p* < 0.001).

#### 3.2.3 ROC Curve Analysis

**FIGURE 3:**
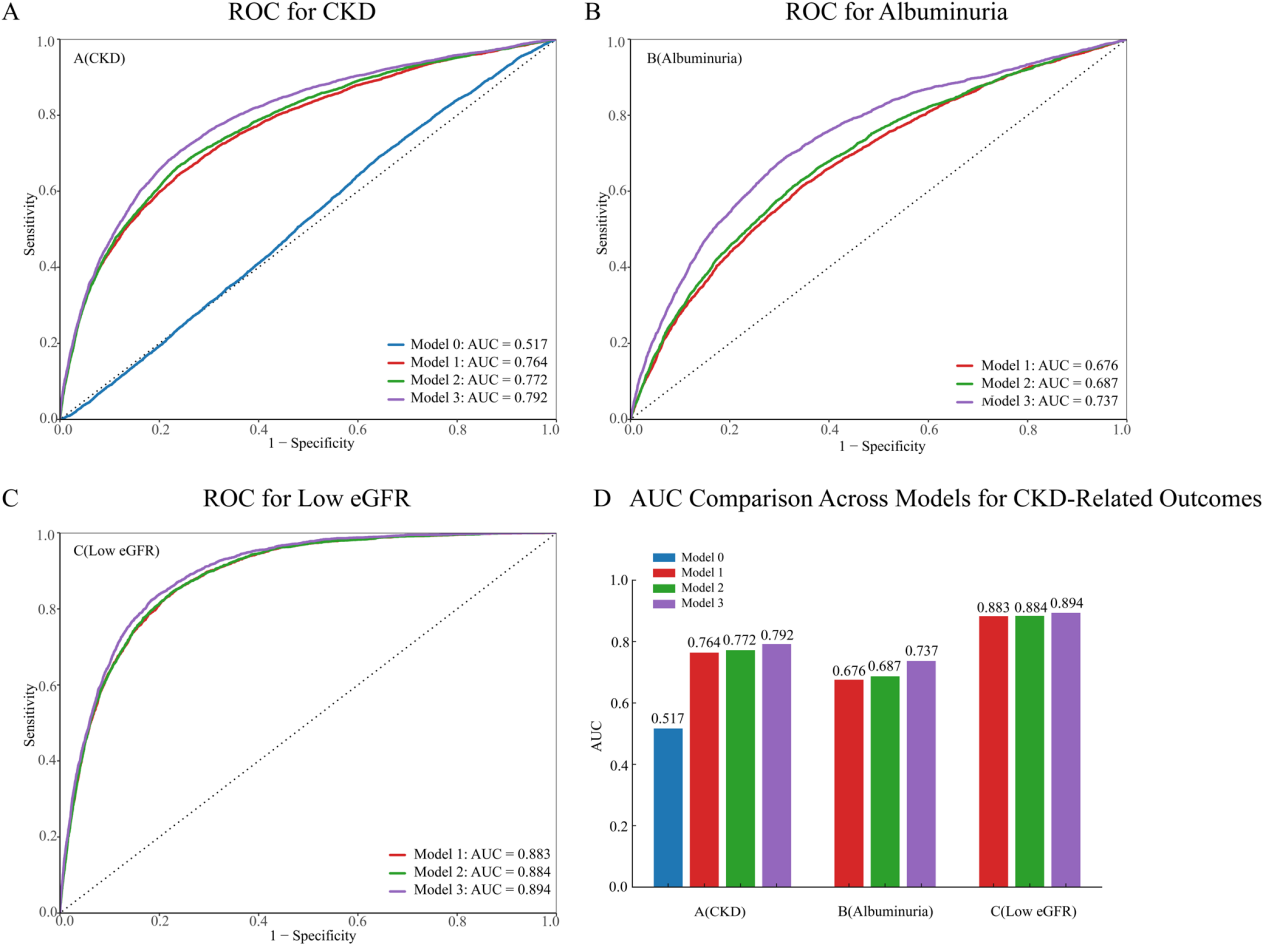
Receiver operating characteristic (ROC) curves for the predictive performance of PHDI in relation to (A) CKD, (B) albuminuria, and (C) low eGFR. (D) Compares the AUC values across the three models for CKD, albuminuria, and low eGFR, showing the improvement in discrimination from Model 1 to Model 3. Model 1 was adjusted for age and sex; Model 2 was further adjusted for race/ethnicity, education level, income level, BMI category, and smoking status; and Model 3 was additionally adjusted for hypertension, diabetes, and hypercholesterolemia. AUC, area under the curve; PHDI, planetary health diet index; CKD, chronic kidney disease; eGFR, estimated glomerular filtration rate.

We used ROC analysis to assess how well the survey‐weighted multivariable logistic models discriminated individuals with prevalent CKD from those without CKD. As shown in Figure 3A, in the unadjusted model containing only PHDI (Model 0), discrimination was poor, with an AUC of 0.517 (95% CI 0.492–0.542). After adding age and sex (Model 1), the AUC increased substantially to 0.764 (95% CI 0.749–0.779). Incorporating sociodemographic variables (Model 2) provided a modest additional improvement (AUC = 0.772; 95% CI 0.757–0.787). The fully adjusted model (Model 3), which further included clinical and lifestyle factors, achieved the highest discrimination (AUC = 0.792; 95% CI 0.777–0.807).

#### 3.2.4 Association Between PHDI and Kidney Function Indicators

We assessed the association between the PHDI and kidney function indicators with survey‐weighted multivariable logistic regression (Table 3). In the fully adjusted specification (Model 3), each one‐point increment in PHDI corresponded to lower odds of impaired kidney function (eGFR < 60 mL/min/1.73 m^2^; OR = 0.986, 95% CI 0.983–0.990; *p* < 0.001), equivalent to an approximate 1.4% decrease in the odds per point. Similar protective trends were observed in Models 1 and 2. In categorical analyses, participants with higher PHDI also had reduced odds of impaired kidney function (OR = 0.761, 95% CI 0.695–0.834; *p* < 0.001).

For albuminuria (ACR ≥ 30 mg/g), each additional PHDI point was associated with a 0.6% reduction in the odds (OR = 0.994, 95% CI 0.991–0.997; *p* < 0.001). Similar protective trends were observed in Models 1 and 2. The high‐PHDI group likewise showed lower odds relative to the low‐PHDI group (OR = 0.860, 95% CI 0.800–0.924; *p* < 0.001).

ROC analyses for impaired kidney function and albuminuria indicated that fully adjusted models—encompassing sociodemographic, clinical, and lifestyle covariates—achieved adequate discriminative performance. As shown in Figure 3B, the discriminative performance for albuminuria improved progressively with model adjustment. Model 1 achieved an AUC of 0.676, which increased slightly after incorporating sociodemographic variables in Model 2 (AUC = 0.687). The fully adjusted Model 3, which additionally accounted for clinical and lifestyle factors, achieved the highest discrimination (AUC = 0.737), indicating a modest yet meaningful enhancement in predictive performance. Figure 3C illustrates the ROC curves for low eGFR (eGFR < 60 mL/min/1.73 m^2^). Across all models, discrimination remained consistently strong. The AUCs were 0.883 for Model 1, 0.884 for Model 2, and 0.894 for the fully adjusted Model 3, demonstrating a high and stable predictive capability for impaired kidney function regardless of covariate adjustment.

Figure 3D summarizes the AUC values across all three outcomes—prevalent CKD, albuminuria, and low eGFR—highlighting the incremental improvement in model discrimination with progressive adjustment. The fully adjusted Model 3, which incorporated clinical and lifestyle factors, achieved the highest AUCs across all outcomes, indicating that broader covariate adjustment enhanced the overall predictive performance of the PHDI‐based models.

#### 3.2.5 PHDI Subcomponents and CKD Prevalence

Component‐level analyses indicated that higher PHDI component scores for legumes (OR = 0.953, 95% CI 0.935–0.972; *p* < 0.001), non‐starchy vegetables (OR = 0.980, 95% CI 0.970–0.990; *p* < 0.001), and nuts (OR = 0.987, 95% CI 0.978–0.996; *p* = 0.006) were linked to lower odds of prevalent CKD (Figure S1). In contrast, higher scores for dairy (OR = 1.029, 95% CI 1.018–1.041; *p* < 0.001), poultry (OR = 1.020, 95% CI 1.012–1.028; *p* < 0.001), saturated oils and trans fats (OR = 1.006, 95% CI 1.001–1.012; *p* = 0.008), and red/processed meat (OR = 1.009, 95% CI 1.001–1.016; *p* = 0.022) corresponded to higher odds of prevalent CKD.

Unexpectedly, fruit scores were positively associated with prevalent CKD (OR = 1.011, 95% CI 1.004–1.019; *p* = 0.003), possibly reflecting the adverse effects of excessive intake of high‐sugar fruits or processed fruit products. Further research is needed to clarify this finding. The other PHDI components were not significantly associated with prevalent CKD, and the complete set of component‐level estimates is displayed in the forest plot (Figure S1).

### 3.3 Association Between PHDI and Mortality

#### 3.3.1 Kaplan–Meier Analysis

**FIGURE 4:**
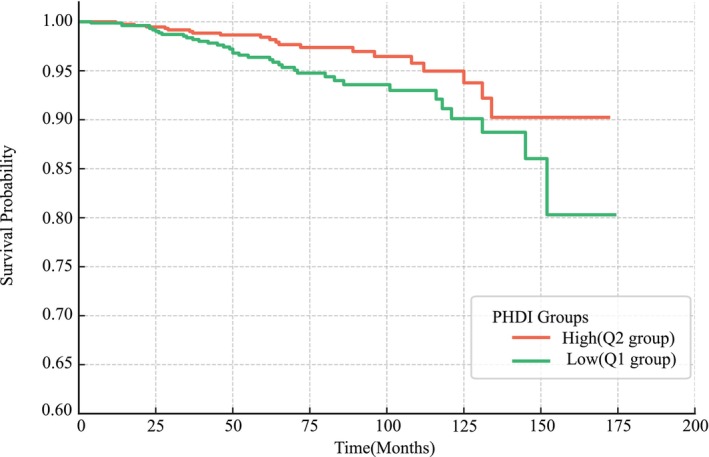
Kaplan–Meier curves for all‐cause mortality among CKD patients according to PHDI score. Participants were dichotomized by the median PHDI (63.2): Q1 (≤ 63.2, lower PHDI) vs. Q2 (> 63.2, higher PHDI). Participants with higher PHDI scores (Q2) had significantly lower mortality risk than those with lower scores (Q1) (log‐rank *p* < 0.001). CKD, chronic kidney disease; PHDI, Planetary Health Diet Index.

Kaplan–Meier survival curves stratified by PHDI score showed higher overall survival among participants with CKD in the higher‐PHDI group, with curve separation beginning around 40 months and widening thereafter (log‐rank *p* < 0.001; Figure 4).

#### 3.3.2 Cox Proportional Hazards Results

During a median follow‐up of 58 months, we documented 1676 deaths from any cause, of which 501 were cardiovascular, 312 were cancer‐related, and 58 were attributed to CKD (Table 4). In the fully adjusted Cox model (Model 3), participants in the higher‐PHDI group (Q2 vs. Q1) had lower CKD‐related mortality (HR = 0.955, 95% CI 0.929–0.981; *p* < 0.001). In contrast, the associations with all‐cause mortality (HR = 0.997, 95% CI 0.993–1.002; *p* = 0.267), cardiovascular mortality (HR = 0.996, 95% CI 0.987–1.005; *p* = 0.394), and cancer mortality (HR = 0.999, 95% CI 0.983–1.015; *p* = 0.878) were not statistically significant. The protective association for CKD‐related mortality was already evident in the intermediate adjustment models (Models 1–2) and remained stable after full covariate control.

**TABLE 4:** Weighted Cox proportional hazards regression analysis on the associations between the Planetary Health Diet Index (PHDI) and all‐cause, CKD‐related, CVD, and cancer mortality.

	All‐cause mortality		CKD‐related mortality		CVD mortality		Cancer mortality	
	HR (95% CI)	*p*	HR (95% CI)	*p*	HR (95% CI)	*p*	HR (95% CI)	*p*
Model 1								
Q1	ref		ref		ref		ref	
Q2	0.997 (0.992, 1.001)	0.136	0.955 (0.931, 0.979)	**< 0.001**	0.996 (0.986, 1.005)	0.333	0.998 (0.984, 1.013)	0.811
Model 2								
Q1	ref		ref		ref		ref	
Q2	1.000 (0.995, 1.004)	0.921	0.958 (0.933, 0.984)	**0.0016**	0.996 (0.987, 1.006)	0.447	0.998 (0.981, 1.014)	0.760
Model 3								
Q1	ref		ref		ref		ref	
Q2	0.997 (0.993, 1.002)	0.267	0.955 (0.929, 0.981)	**< 0.001**	0.996 (0.987, 1.005)	0.394	0.999 (0.983, 1.015)	0.878

*Note:* Model 1: Adjusted for age and sex. Model 2: Additionally adjusted for race/ethnicity, education level, income level, BMI category, and smoking status. Model 3: Additionally adjusted for hypertension, diabetes, and hypercholesterolemia. PHDI was analyzed as a binary variable (Q2 vs. Q1). Results are presented as hazard ratios (HRs) with 95% confidence intervals (CIs). Bold indicates *p*‐value < 0.05.

Abbreviations: CI, confidence interval; CKD, chronic kidney disease; HR, hazard ratio; PHDI, planetary health diet index.

#### 3.3.3 Nonlinear Association Between PHDI and CKD‐Related Mortality

Generalized additive and restricted cubic spline analyses identified a nonlinear pattern for CKD‐related mortality. Figure 2C displays the RCS results for the association between PHDI and CKD‐related mortality. The test for nonlinearity (*p* for nonlinear = 0.037) indicated a significant departure from a simple linear relationship, suggesting the presence of a potential threshold. The overall association was statistically significant (*p* for overall < 0.001), supporting the need for further evaluation using segmented regression to locate the breakpoint.

As shown in Figure 2D, breakpoint (segmented) Cox regression confirmed an inflection at PHDI = 47. The analysis showed no statistically meaningful trend below this threshold (HR = 1.056, 95% CI 0.881–1.262; *p* = 0.547). Conversely, PHDI scores above 47 were associated with a significant inverse association (HR = 0.947, 95% CI 0.921–0.982; *p* < 0.001), consistent with the notion of a dietary threshold influencing CKD‐related mortality risk.

#### 3.3.4 Time‐Dependent ROC Analysis

**FIGURE 5:**
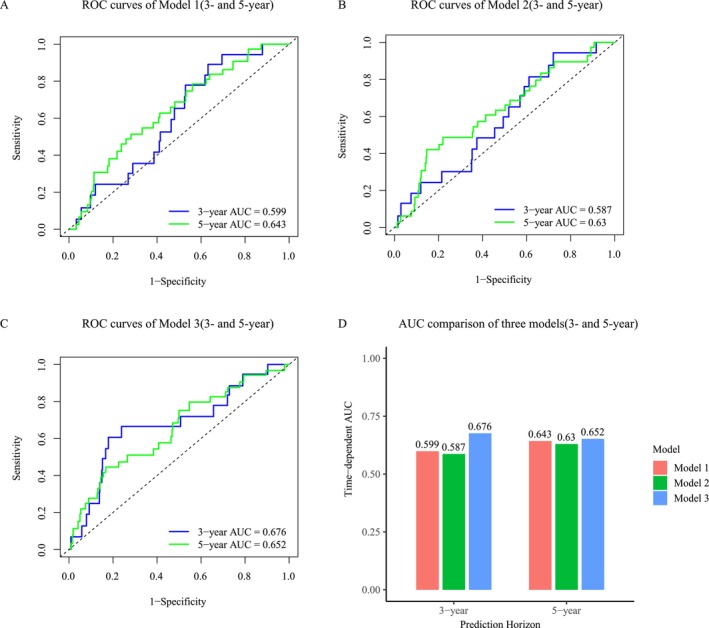
Time‐dependent ROC curves and AUC comparison for the prediction of CKD‐related mortality among CKD patients. (A–C) ROC curves for Models 1–3 at 3‐ and 5‐year follow‐up. (D) Comparison of AUC values across the three models at 3 and 5 years. Model 1 was adjusted for age and sex; Model 2 was further adjusted for race/ethnicity, education level, income level, BMI category, and smoking status; and Model 3 was additionally adjusted for hypertension, diabetes, and hypercholesterolemia. AUC, area under the curve; CKD, chronic kidney disease.

Time‐dependent receiver operating characteristic analyses were used to assess discrimination for CKD‐related mortality. As shown in Figure 5A,B and, the AUCs for Model 1 were 0.599 and 0.643 at the 3‐year and 5‐year horizons, respectively, while Model 2 yielded AUCs of 0.587 and 0.630. Figure 5C presents the fully adjusted Model 3, which achieved AUCs of 0.676 at 3 years and 0.652 at 5 years. Figure 5D summarizes these results, demonstrating that Model 3 consistently provided the best discriminative performance across both prediction horizons.

#### 3.3.5 Association Between PHDI Subcomponents and CKD‐Related Mortality

In the fully adjusted Cox model (Model 3), among the 14 PHDI components, only the nuts component score was inversely associated with CKD‐related mortality (HR = 0.841, 95% CI 0.719–0.984; *p* = 0.031). No other PHDI components showed statistically significant associations with CKD‐related mortality.

## 4 Discussion

We investigated associations between the PHDI and the outcomes of chronic kidney disease, including its prevalence and mortality. Higher PHDI adherence was associated with a significant attenuation of CKD risk, and this protective association increased among participants with higher PHDI scores. These findings align with prior evidence that plant‐forward, high‐fiber dietary patterns support CKD prevention (Hu et al. 2019; Naber and Purohit 2021; Cigarrán Guldris et al. 2022). This association highlights the clinical significance of promoting sustainable dietary patterns that emphasize increased intake of plant‐based components for kidney health.

In terms of baseline characteristics, the unadjusted mean PHDI score was slightly higher among participants with CKD, possibly because of age‐related patterns: in our sample, older individuals tended to have higher PHDI scores, and advanced age is a well‐established risk factor for CKD (GBD Chronic Kidney Disease Collaboration 2020; Stevens et al. 2024). The combination of these two age‐linked trends produces the apparent “paradox” observed in the unadjusted comparison. In subsequent analyses, after age and sex were removed as key confounders, the association shifted in a biologically plausible direction, with higher PHDI scores demonstrating a consistent protective effect against CKD.

This study revealed a nonlinear relationship between the PHDI and CKD, with improvements in diet quality significantly reducing CKD prevalence risk for PHDI scores exceeding 64 (OR = 0.987, *p* < 0.001). Additionally, a lower risk of CKD‐related mortality emerged when the PHDI score exceeded 47 (HR = 0.947, 95% CI 0.921–0.982; *p* < 0.001). Studies have indicated that the protective effect of diet quality becomes evident only after a certain threshold is reached (Hu et al. 2019; Soltani et al. 2019; Trichopoulou et al. 2003). This nonlinear relationship provides specific guidance for dietary interventions, emphasizing the need to surpass a minimum threshold to achieve protective effects.

We found an inverse association between the PHDI and albuminuria, which aligns with prior findings that kidney‐friendly dietary patterns can slow CKD progression and reduce albuminuria (Apetrii et al. 2021). These findings suggest that improved diet quality may help mitigate kidney damage and prevent early‐stage kidney functional decline (Tang, Zhang, et al. 2025; Apetrii et al. 2021; Rysz et al. 2017). This study also indicates that among PHDI subcomponents, the intake of legumes, nuts, and non‐starchy vegetables is associated with lower CKD prevalence. Notably, nut consumption not only affects CKD prevalence but also significantly correlates with reduced CKD‐related mortality. These findings support the protective effects of healthy fatty acids, antioxidants, and fiber found in nuts (Rysz et al. 2021; Moldovan et al. 2024; Ros 2010).

On comparing the effects of following the PHDI with those of other dietary patterns, we found that consumption of a Mediterranean diet positively influences kidney function in CKD patients (Gallieni and Cupisti 2016; D'Alessandro et al. 2023; Charkviani et al. 2023), and that Dietary Approaches to Stop Hypertension (DASH)‐style dietary patterns may help to improve blood pressure control and reduce the risk of CKD onset and progression, partly through lower sodium and higher potassium intakes (Gallieni and Cupisti 2016; Tyson et al. 2016; Ajjarapu et al. 2019). However, the PHDI appears to deliver distinct and more durable benefits, as it is related to a lower CKD prevalence and a significant reduction in CKD‐related mortality. Furthermore, the PHDI was explicitly developed as a sustainability‐focused dietary index and has been shown to align higher scores with lower diet‐related greenhouse gas emissions (Willett et al. 2019; Cacau et al. 2021; Frank, Jaacks, Meyer, et al. 2024). Emerging evidence has further associated higher PHDI adherence with slower cognitive decline and improved psychological well‐being in older adults (Frank, Jaacks, Avery, et al. 2024; Tang, Yu, et al. 2025; Jiang et al. 2025). The PHDI may thus serve as a more comprehensive tool for evaluating the impact of diet on both individual and planetary health.

The innovation of this study lies in its novel use of the PHDI—the first dietary index that simultaneously integrates individual health and environmental sustainability—to investigate its nonlinear association with CKD prevalence, its relationships with a low eGFR and albuminuria levels, and its association with CKD‐related mortality and threshold effects. In addition, this study analyzes the independent associations of PHDI subcomponents with CKD risk, aiming to provide practical and feasible guidance for CKD prevention and management from the perspective of sustainable nutrition.

Despite its strengths, including extensive adjustment for confounding variables and the use of large‐scale NHANES data, this study has several limitations. First, 24‐h dietary recalls may not fully capture individuals' customary intake, and the NHANES does not include repeated dietary assessments over time; therefore, potential changes in dietary habits during the follow‐up period could not be evaluated, but the use of NHANES survey weights allowed us to obtain nationally representative estimates of PHDI scores (Centers for Disease Control and Prevention (CDC) 2017; Centers for Disease Control and Prevention (CDC) 2018). Second, supplement use was not considered, and as the dietary data were self‐reported, reporting bias is possible. Third, although broad adjustment was performed for confounding variables, residual confounding from unmeasured lifestyle or clinical factors cannot be excluded. Finally, the number of CKD‐related deaths was relatively small, which may limit statistical power and require cautious interpretation of mortality‐related results.

## 5 Conclusion

Adherence to the PHDI was inversely related to CKD prevalence and CKD‐related mortality, particularly at higher scores. The component analysis highlighted consumption of nuts, non‐starchy vegetables, and legumes as robust protective indicators. These findings support the use of PHDI‐aligned counseling as a feasible strategy for CKD prevention and management while motivating studies on underlying mechanisms and cross‐population applicability of this counseling.

## Author Contributions

**Yufei Ming:** software, writing – original draft, data curation. **Chen Qu:** writing – review and editing, funding acquisition. **Zhonghua Sun:** writing – original draft; Data curation. **Minyi Tao:** writing – review and editing; Investigation. **Jiahui Yang:** writing – review and editing; Investigation. **Shuyan Li:** writing – review and editing; Validation. **Xinyu Tao:** writing – review and editing; Software. **Haiyang Wang:** writing – review and editing; Software. **Zhengxia Liu:** writing – review and editing; Funding acquisition.

## Funding

This study was supported by Jiangsu Provincial Elderly Health Research Key Project (Grant No. LKZ2024005) and the Natural Science Foundation of Jiangsu Province (Grant No. BK20242002).

## Ethics Statement

This secondary analysis of the NHANES study did not require additional ethical review, as it had already received approval from the National Center for Health Statistics Institutional Review Board and was carried out with informed consent (https://www.cdc.gov/nchs/nhanes/irba98.htm).

## Conflicts of Interest

The authors declare no conflicts of interest.

## Supporting information


**Table S1:** Calculation of the Planetary Health Diet Index (PHDI).
**Figure S1:** Associations of PHDI components with CKD prevalence. Forest plot displaying adjusted odds ratios (ORs) and 95% CIs for each dietary component. Green indicates protective associations (OR < 1, *p* < 0.05), red indicates harmful associations (OR > 1, *p* < 0.05), and gray denotes non‐significant results.

## Data Availability

The data that support the findings of this study are openly available in National Health and Nutrition Examination Survey (NHANES) at https://www.cdc.gov/nchs/nhanes/.
