# Supplementary material for: Association Between the Planetary Health Diet Index and Chronic Kidney Disease Prevalence and Mortality: An Analysis of NHANES 2005–2018
Source: Food Sci Nutr. 2025 Dec 26;14(1):e71402. doi: 10.1002/fsn3.71402 (PMC12743143; doi:10.1002/fsn3.71402)
Supplement: Supplementary file 1 — Table S1: Calculation of the Planetary Health Diet Index (PHDI). Figure S1: Associations of PHDI components with CKD prevalence. Forest plot displaying adjusted odds ratios (ORs) and 95% CIs for each dietary component. Green indicates protective associations (OR < 1, p < 0.05), red indicates harmful associations (OR > 1, p < 0.05), and gray denotes non‐significant results. [file FSN3-14-e71402-s001.docx]

**Supplementary Material**

| **Table S1.** Calculation of the Planetary Health Diet Index (PHDI). | | |
| --- | --- | --- |
| **Dietary component** | **Category minimum score** (0 points) | **Category maximum score** (10 points) |
| ***Adequacy components*** | | |
| Whole grains^1^ | 0 grams | ≥ 75 grams for women  ≥ 90 grams for men |
| Whole fruits (excludes fruit juice) | 0 grams | ≥ 200 grams |
| Non-starchy vegetables | 0 grams | ≥ 300 grams |
| Nuts and seeds | 0 grams | ≥ 50 grams |
| Legumes |  |  |
| Non-soy legumes^2,3^ | 0 grams | 100 grams |
| Soybean/ soy foods^2,3^ | 0 grams | 50 grams |
| Unsaturated oils | 0% of total energy intake | ≥ 10% of total energy intake |
| ***Moderation components*** | | |
| Starchy vegetables | ≥ 200 grams | ≤ 50 grams |
| Dairy^4^ | ≥ 4.08 cup-equivalents | ≤ 1.02 cup-equivalents |
| Red and processed meat | ≥ 300 grams | ≤ 14 grams |
| Poultry | ≥ 58 grams | ≤ 29 grams |
| Eggs | ≥ 120 grams | ≤ 12 grams |
| Fish | ≥ 50 grams | ≤ 15 grams |
| Saturated oils and trans fats | ≥ 21% of total energy intake | ≤ 3.5% of total energy intake |
| Added sugar and fruit juice | ≥ 25% of total energy intake | ≤ 5% of total energy intake |
| ^1^Thresholds were anchored to the midpoints of the intake ranges recommended in the EAT-Lancet Commission Scientific Report.  ^2^Intakes (g/day) were computed on a dry-weight basis.  ^3^For the legumes component, soy and non-soy subcomponents were each assigned a weight of 0.5 in score calculation.  ^4^In FPED, one dairy serving equals 245 g of whole milk or an equivalent product. Per the EAT-Lancet scheme, the maximum score corresponds to ≤250 g whole milk (or equivalent) and the minimum score to ≥1000 g whole milk (or equivalent).  ^5^ “Unsaturated oils” and “saturated oils and trans fats” are nutrient-based components calculated as their percentage contribution to total energy intake.  Note: Dietary component amounts were derived from the Food Patterns Equivalents Database (FPED) based on 24-h dietary recall data (https://www.ars.usda.gov/northeast-area/beltsville-md-bhnrc/beltsville-human-nutrition-research-center/food-surveys-research-group/docs/fped-databases/). | | |


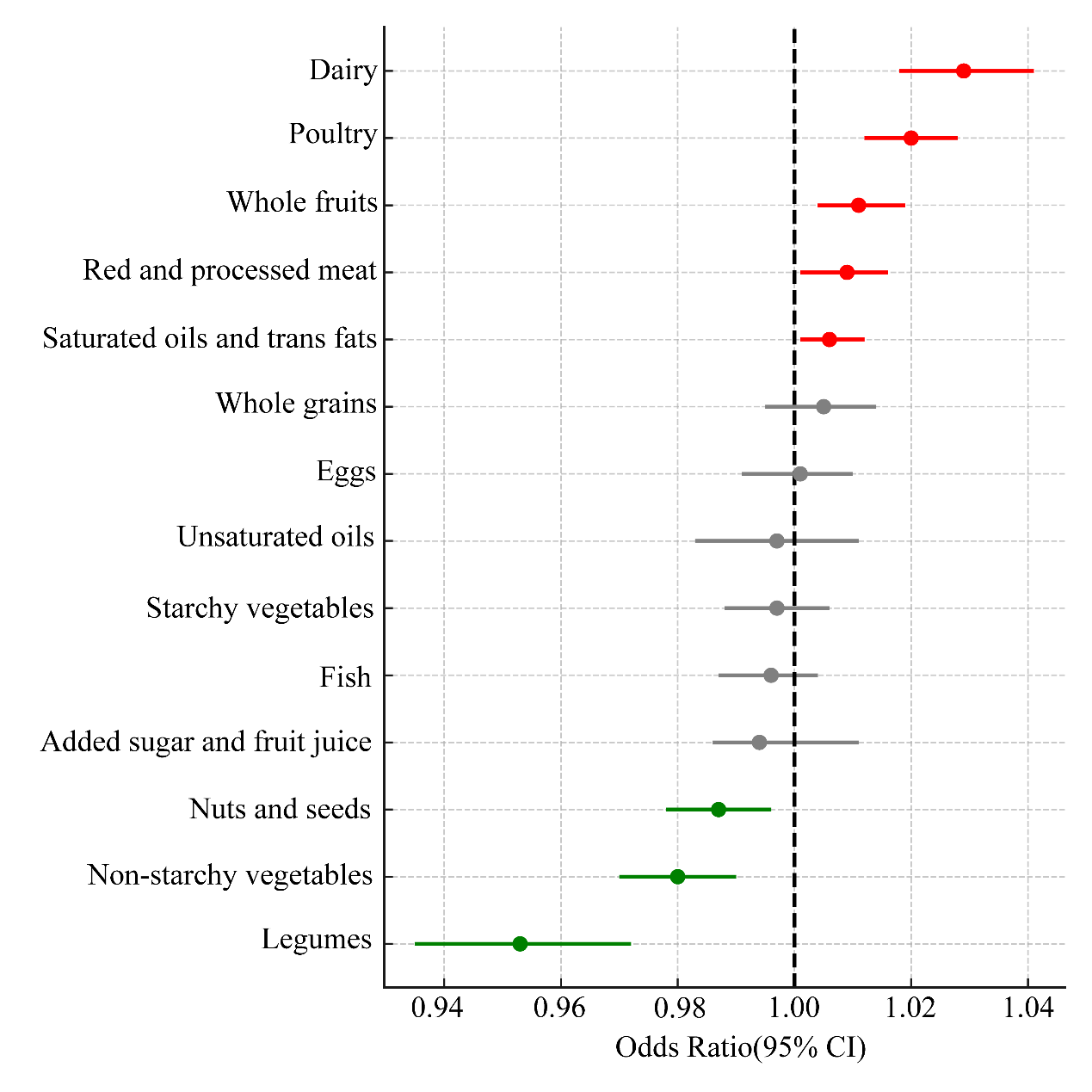


Fig. S1. Associations of PHDI components with CKD prevalence.Forest plot displaying adjusted odds ratios (ORs) and 95% CIs for each dietary component. Green indicates protective associations (OR < 1, *p* < 0.05), red indicates harmful associations (OR > 1, *p* < 0.05), and grey denotes non-significant results.
